# *Acanthamoeba* spp. as Factors for Severe Infectious Diseases in Humans

**DOI:** 10.3390/microorganisms12030581

**Published:** 2024-03-14

**Authors:** Lidia Chomicz, Jacek P. Szaflik, Wanda Baltaza

**Affiliations:** 1 Department of Medical Biology, Medical University of Warsaw, 00-575 Warsaw, Poland; 2 Department of Ophthalmology, Independent Public Clinical Ophthalmology Hospital, Medical University of Warsaw, 00-576 Warsaw, Poland; 3 Department of Public Health, Medical University of Warsaw, 02-097 Warsaw, Poland; wanda.baltaza@wum.edu.pl

*Acanthamoeba* spp., known as free-living organisms, occur in both natural and man-made habitats, including those in soil, air, water bodies of varying types, in health facilities, hospital environments, and on/in equipment. These heterotrophic organisms exist as trophozoites which feed on bacteria and as dormant cysts. Previously, *Acanthamoeba* spp. were considered to be harmless exozoic protists that develop without entering the human body; However, infections caused in humans by *Acanthamoeba* were reported in the 1960s and 1970s [1,2,3,4]. Frequent exposure to the amoebae result in the occurrence of IgG anti-*Acanthamoeba* antibodies in non-infected persons. Non-specific symptoms caused by *Acanthamoeba* spp., and endondosymbionts surviving in them, affect the diagnosis correctness of [4,5,6,7,8]. 

*Acanthamoeba* spp. present a serious risk for human health as causative factors of encephalitis and keratitis; the amoebae can also cause cutaneous and nasopharyngal infections. 

## 1. Granulomatous Amoebic Encephalitis (GAE)

Granulomatous amoebic encephalitis (GAE), a rare but usually fatal disease exhibiting neurological symptoms resembling viral or bacterial meningitis, is the subject of epidemiology, physiology, diagnosis, and treatment studies [1,3,6,7,8]. A loss of consciousness and coma both result in a poor prognosis. GAE symptoms common to other of CNS diseases can cause misdiagnosis; magnetic resonance imaging and computed tomography are helpful for this reason. GAE develops in persons with HIV/AIDS and those who are immunocompromised or undergoing immunosuppressive therapy [8,9,10]. The amebae enter the human body through the nose and into to bloodstream, crossing blood–brain barrier and invade the CNS; infections via skin lesions or the respiratory tract are also likely. Complex therapy (ketoconazole, azithromycin, and pentamidine isethionate) is less effective due to the resistance of *Acanthamoeba* to chemicals and the inability of drugs to cross the blood–brain barrier [3,8,10]. Despite some success in treatment efforts, the prognosis for GAE is still poor. 

## 2. *Acanthamoeba* Keratitis (AK)

*Acanthamoeba* keratitis (AK) initially shows non-specific and confusing clinical symptoms similar to viral, fungal, or bacterial keratitis: ring-shaped cornea infiltrations, characteristic of AK, occurs in ~50% of cases. It is considered a rare but devastating infection that occurs mainly in immunocompetent persons, and it is associated with a high risk of blindness. The main risk factor for this type of infection is poor hygiene when wearing contact lenses; micro-injuries to the cornea and foreign bodies in the eyes predispose people to AK [8,10,11]. The binding of amoebae to contact lenses and the cornea is the first step in the development of AK, which is usually unilateral. The use of a slit-lamp, in vivo confocal microscopy, direct microscopic examinations, in vitro cultures, and molecular techniques are common in related research and in medical practice to detect causative agents and assess pathological effects [11,12,13]. The recommended drugs for this infection belong to the diamides and biguanides groups. The presence of cysts in the eye despite treatment creates the risk of the emergence of trophozoites and AK recrudescence. Anti-adhesive agents are investigated with contact-lens solutions that are not effective enough against the amoebae [8,13,14,15]. Non-specific AK symptoms are common causes of misdiagnosis and ineffective therapy. 

## 3. Conclusions

Infections caused by *Acanthamoeba* strains posing a public-health threat worldwide are detected and reported with constantly increasing frequency. 

*Microorganisms* is one of the leading journals in this area of research, in which the Special Issue 

“*Acanthamoeba* spp. as Factors for Severe Infectious Diseases in Humans” includes papers on the serious infections caused in people by amphizoic amoebae. The publications in this Special Issue cover an important spectrum of current research subjects: mainly, the problems relating to the fatal granulomatous encephalitis and *Acanthamoeba* keratitis, connected by their associated high risk of visual deterioration. There are many challenges associated with the extreme resistance of *Acanthamoeba* cysts to chemicals. Preventive actions are major factors inhibiting the risk of GAE and AK; however, the ability to inhibit the spread of *Acanthamoeba* infections is limited, due to the common occurrence of the amoebae in various environments. 

There is an urgent need for further research work to develop effective and reliable *Acanthamoeba* diagnostic tests. 

Future studies will make significant impacts by identifying novel agents/drugs that are able to cross the blood–brain barrier and will be extremely important for progression in terms of treatment efficacy.

Innovative concepts in the search for anti-adhesive agents are needed to improve the prevention of amoebic infection in humans. 

Taking proper care in the handling of contact lenses is extremely important for the reasons we have described. 

Moreover, advanced research is needed in order to enhance the anti-adhesive activity of contact-lens solutions. 

Further studies are also needed to examine the ability of amphizoic amoebae in dispersing endosymbionts that are pathogenic to humans.
